# Comparative Analysis of Gene Content Evolution in Phytoplasmas and Mycoplasmas

**DOI:** 10.1371/journal.pone.0034407

**Published:** 2012-03-27

**Authors:** Ling-Ling Chen, Wan-Chia Chung, Chan-Pin Lin, Chih-Horng Kuo

**Affiliations:** 1 Institute of Plant and Microbial Biology, Academia Sinica, Taipei, Taiwan; 2 Department of Plant Pathology and Microbiology, National Taiwan University, Taipei, Taiwan; Columbia University, United States of America

## Abstract

Phytoplasmas and mycoplasmas are two groups of important pathogens in the bacterial class Mollicutes. Because of their economical and clinical importance, these obligate pathogens have attracted much research attention. However, difficulties involved in the empirical study of these bacteria, particularly the fact that phytoplasmas have not yet been successfully cultivated outside of their hosts despite decades of attempts, have greatly hampered research progress. With the rapid advancements in genome sequencing, comparative genome analysis provides a new approach to facilitate our understanding of these bacteria. In this study, our main focus is to investigate the evolution of gene content in phytoplasmas, mycoplasmas, and their common ancestor. By using a phylogenetic framework for comparative analysis of 12 complete genome sequences, we characterized the putative gains and losses of genes in these obligate parasites. Our results demonstrated that the degradation of metabolic capacities in these bacteria has occurred predominantly in the common ancestor of Mollicutes, prior to the evolutionary split of phytoplasmas and mycoplasmas. Furthermore, we identified a list of genes that are acquired by the common ancestor of phytoplasmas and are conserved across all strains with complete genome sequences available. These genes include several putative effectors for the interactions with hosts and may be good candidates for future functional characterization.

## Introduction

Phytoplasmas and mycoplasmas are two groups of important pathogenic bacteria in the class Mollicutes [1]–[5]. Recent large-scale phylogenetic studies using available genome sequences suggested that Mollicutes form a monophyletic clade and are closely related to lineages in the phylum Firmicutes, such as Bacilli and Clostridia [6], [7]. Compared to these related lineages that maintain a free-living lifestyle, the parasitic phytoplasmas and mycoplasmas all have highly reduced genomes and limited metabolic capacities. For example, the tricarboxylic acid cycle, oxidative phosphorylation, nucleotide biosynthesis, fatty acids biosynthesis, and the biosynthesis of most amino acids all appear to have been disrupted in these bacteria [8]–[15].

However, despite the close evolutionary relationship and the similarities in their parasitic lifestyles, phytoplasmas and mycoplasmas differ in several aspects. While phytoplasmas are insect-transmitted plant pathogens, mycoplasmas are restricted to vertebrate hosts. In addition, mycoplasmas have adapted an alternative genetic code that uses the codon UGA for the amino acid tryptophan instead of the usual opal stop codon [16]. Finally, although mycoplasmas can be cultured in the laboratory and are amenable to genetic manipulations [17], cultivation of phytoplasma cells outside of the host has remained as an unresolved challenge [5]. The inability to maintain phytoplasmas in pure cultures has resulted in the designation of ‘*Candidatus*’ status in their taxonomic assignment [18] and also greatly hampered the efforts to study these plant pathogens despite their worldwide economical importance [19].

With the recent advancements in genomics, the complete genome sequences from several phytoplasma species have become available and these data sets have provided an unprecedented opportunity to understand their genetic makeup [8]–[11], [20], [21]. Furthermore, as the number of available genome sequences increases, it becomes possible to utilize a comparative approach based on a phylogenetic framework to investigate the evolution of gene content in the lineages of interest [22]–[24].

In this study, we focus on the inference of gene gains and losses in phytoplasmas, mycoplasmas, and their common ancestor. By incorporating two suitable outgroups, the class Bacilli (represented by *Bacillus subtilis* [25] and *Lactobacillus plantarum* [26]) and the class Clostridia (represented by *Clostridium kluyveri* [27] and *Pelotomaculum thermopropionicum* [28]), we are able to establish the ancestral state of gene presence or absence in the common ancestor of Mollicutes. Additionally, because *Bacillus subtilis* is an important model organism for molecular genetic studies, its genome sequence and protein coding genes are well annotated [25], [29], [30] and are useful for inferring the functional significance of homologous genes in related species. Taken together, with a combination of appropriate taxon sampling, large-scale comparative analysis, and careful examination of the results, our findings provide insights into the history of gene content evolution in Mollicutes.

## Results

### Organismal phylogeny and core genes

The annotations provided in the GenBank records include a total of 19,462 protein coding sequences from the 12 genomes examined in this study (Table 1). Our homologous gene identification procedure inferred 10,508 homologous gene clusters (Table S1), including 7,384 singletons. These singletons are clusters that contain a single gene without any homolog, which are specific to an individual genome by definition. On average, approximately 20% of the genes in the phytoplasma genomes and 31% of the genes in the mycoplasma genomes were classified as singletons. These proportions are substantially lower than that found in the four outgroup genomes (average = 42%), suggesting that this type of genes may have been preferentially lost during the reductive genome evolution in Mollicutes.

**Table 1:** List of the genome sequences included in this study.

Genome	RefSeq	Size (Mb)	% GC	% coding	No. of CDS^a^	% without homolog
‘*Ca.* Phytoplasma asteris’ AYWB [8]	NC_007716	0.71	26	73	671	28
‘*Ca.* Phytoplasma asteris’ OY-M [9]	NC_005303	0.85	27	72	750	20
‘*Ca.* Phytoplasma australiense’ [10]	NC_010544	0.88	27	64	684	16
‘*Ca.* Phytoplasma mali’ [11]	NC_011047	0.60	21	76	479	17
*Mycoplasma agalactiae* [12]	NC_013948	1.01	29	87	813	26
*Mycoplasma mobile* [13]	NC_006908	0.78	24	90	633	31
*Mycoplasma genitalium* [14]	NC_000908	0.58	31	90	475	33
*Mycoplasma mycoides* [15]	NC_005364	1.21	23	81	1,017	36
*Bacillus subtilis* [25]	NC_000964	4.22	43	87	4,176	46
*Lactobacillus plantarum* [26]	NC_004567	3.31	44	83	3,007	39
*Clostridium kluyveri* [27]	NC_009706	3.96	32	84	3,919	42
*Pelotomaculum thermopropionicum* [28]	NC_009454	3.03	52	85	2,977	42

a Number of protein coding sequences.

**Figure 1:**
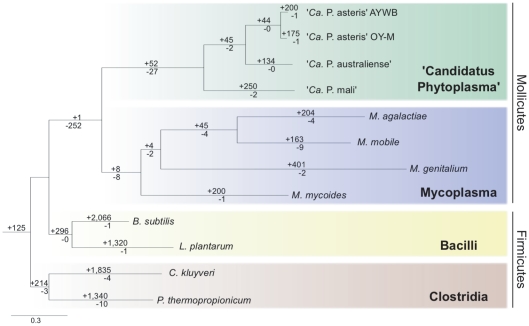
Organismal phylogeny and distribution of lineage-specific gene clusters. The organismal phylogeny is inferred from the concatenated protein alignment of 105 single-copy genes shared by all lineages (with 44,919 aligned amino acid sites), the three phylogenetic methods used (*i.e.*, maximum likelihood, parsimony, and Bayesian) all produced the same tree topology with strong support (*i.e.*, all internal nodes received 100% bootstrap support using the maximum likelihood method and >97% clade credibility using the Bayesian method). The branch lengthes shown in this figure is based on the maximum likelihood result. The numbers above a branch and proceeded by a ‘+’ sign indicate the number of homologous gene clusters that are uniquely present in all daughter lineages; the numbers below a branch and proceeded by a ‘−’ sign indicate the number of homologous gene clusters that are uniquely absent. For example, 52 gene clusters are shared by all four ‘*Candidatus* Phytoplasma’ genomes and do not contain homolog from any of the other eight genomes analyzed (*i.e.*, represent possible gene gain events in the common ancestor of ‘*Ca.* Phytoplasma’ lineages); similarly, 27 gene clusters are missing from the four ‘*Ca.* Phytoplasma’ genomes but are present in all other eight genomes (*i.e.*, represent possible gene loss events in the common ancestor of ‘*Ca.* Phytoplasma’ lineages).

To determine the evolutionary relationship among these genomes, we selected 105 homologous genes that are present as single-copy genes in all 12 genomes examined for phylogenetic inference. Based on the concatenated alignment of these genes (containing 44,919 aligned amino acid sites), the three phylogenetic methods that we used (*i.e.*, maximum likelihood, parsimony, and Bayesian) all produced the same tree topology (Figure 1). This organismal phylogeny is consistent with our previous understanding of Mollicutes evolution [6], [31]. Furthermore, all internal nodes received 100% bootstrap support in the maximum likelihood analysis and >97% clade credibility in the Bayesian inference.

**Figure 2:**
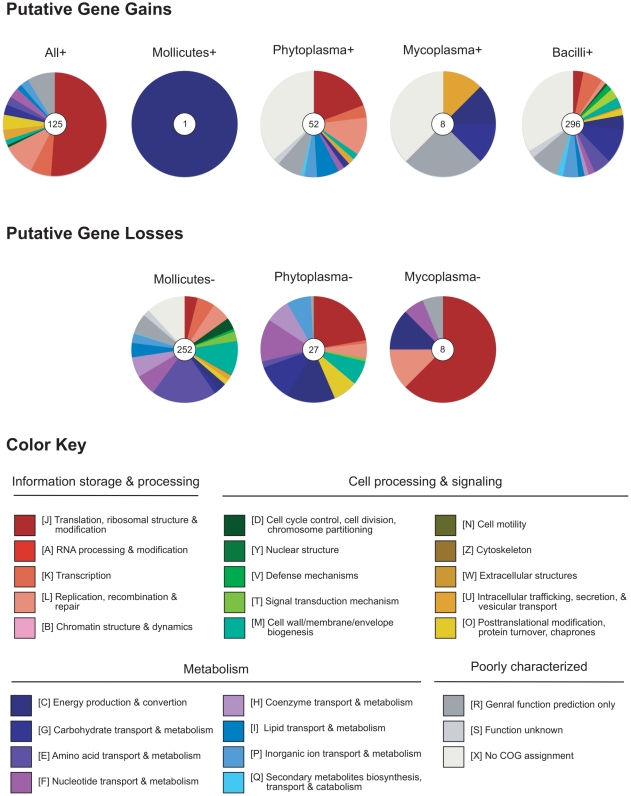
Distribution of COG functional category assignments. The functional categorization of each homologous gene clusters was classified according to the COG assignments, genes that do have any inferred COG annotation were assigned to a custom category X. The numbers in the center of each pie chart indicate the number of homologous gene clusters in each set (*e.g.*, the ‘All+’ set contains 125 homologous gene clusters that are shared by all 12 genomes examined and the ‘Mollicutes−’ set contains 252 homologous gene clusters that are inferred to have been lost in the common ancestor of phytoplasmas and mycoplasmas).

In addition to the 105 single-copy genes used for phylogenetic inference, we found an additional 20 homologous gene clusters that are present in all 12 genomes (with paralogous genes in some of the genomes). Taken together, these 125 homologous genes represent the conserved core gene set among these genomes. On average, these core genes account for approximately 19% of the protein-coding genes in Mollicutes genomes and only approximately 4% in the outgroups. We designated this set of genes as ‘All+’, detailed information about each of the genes in this list is provided in the supplementary material (Table S2). As expected, most of these core genes are essential to cell functions. For example, genes involved in translation, ribosomal structure and biogenesis (COG category J) account for 51% of this gene set (Figure 2). Other important functional categories include DNA replication, recombination and repair (COG category L, 10% of this gene set), transcription (COG category K, 6% of this gene set), and posttranslational modification, protein turnover, and chaperones (COG category O, 5% of this gene set). Notably, we are able to obtain COG functional category assignment for each of the genes in this core gene set and none was assigned as function unknown (COG category S).

### Mollicutes-specific gene gain and losses

Using the organismal phylogeny (Figure 1) as a foundation, we classified the homologous gene clusters according to the pattern of presence and absence in each of the selected genomes. Homologous gene clusters that can be explained by a single gene gain or loss events were counted and mapped on the phylogeny.

For the common ancestor of phytoplasmas and mycoplasmas, we identified only one putative gene gain (*i.e.*, the ‘Mollucutes+’ set in Figure 2 and Table S2), which is an inorganic pyrophosphatase (*ppa*). This enzyme catalyzes the hydrolysis of inorganic pyrophosphate to inorganic phosphate and provides thermodynamic pull for many biosynthetic reactions [32], [33]. It is possible that the acquisition of this gene complimented some of the defects in energy utilization such as the lack of oxidative phosphorylation and the tricarboxylic acid cycle in Mollicutes [9]. Although the outgroups shared a manganese-dependent inorganic pyrophosphatase (*ppaC*), these two genes have no significant sequence similarity and are likely to have independent origins.

In contrast to the paucity of putative gene acquisition, we observed 252 putative gene losses in the common ancestor of phytoplasmas and mycoplasmas. (*i.e.*, the ‘Mollucutes−’ set in Figure 2 and Table S2). Genes involved in amino acid metabolism (COG category E) represent the largest category and account for 20% of this gene set. Notable examples include the biosynthesis of arginine (*argB*, *argC*, *argD*, *argG*, *argH*, *argJ*, and *carB*), histidine (*hisA*, *hisB*, *hisD*, *hisF*, *hisG*, *hisH*, *hisI*, and *hisJ*), lysine/threonine (*asd*, *dapB*, *dapF*, *dapG*, *hom*, *lysA*, *patA*, and *thrB*), proline (*proA*, *proH*, and *proJ*), and aromatic amino acids (*aroA*, *aroB*, *aroE*, *aroF*, *hisC*, *pabA*, *trpA*, *trpB*, *trpC*, *trpD*, *trpE*, and *tyrA*). Furthermore, we also found that genes associated with the biosynthesis of purine (*guaA*, *purC*, *purD*, *purE*, *purF*, *purH*, *purL*, *purM*, and *purN*), pyrimidine (*pyrB*, *pyrC*, *pyrD*, and *pyrR*), thiamine (*thiD*, *thiE*, *thiF*, and *thiN*), isoprenoids (*ipk*, *ispD*, and *uppS*), and fatty acids (*accA*, *accC*, *accD*, *fabD*, *fabF*, *fabHB*, and *fabZ*) all appear to have been lost early in the evolution of Mollicutes.

In addition to the massive losses of biosynthesis pathways for various essential biomolecules as noted above, genes involved in COG category L (replication, recombination and repair) account for 6% of putative losses in the common ancestor of Mollicutes. Notable examples in this category include mismatch repair (*mutL*, *mutS*, and *mutSB*) and double-strand break repair (*recF*, *recN*, and *recO*). The loss of these DNA repair enzymes are commonly observed in other host-dependent bacteria [34] and contributed to the high rates of mutation accumulation in these genomes (see the long branch lengths leading to phytoplasmas and mycoplasmas in Figure 1). Finally, consistent with the lack of cell wall being a defining characteristic of Mollicutes, we identified 27 genes in COG category M (cell wall/membrane/envelop biogenesis) that have been lost and thus disrupting the biosynthesis of two major components of cell wall in Gram-positive bacteria: peptidoglycan (*alr*, *ddl*, *glmS*, *mraY*, *murAA*, *murB*, *murC*, *murD*, *murE*, *murF*, and *murG*) and teichoic acid (*dltB*, *mnaA*, *tagA*, and *tagO*).

### Phytoplasma-specific gene gains and losses

For the common ancestor of the four phytoplasma lineages examined, our phylogenetic approach identified 52 putative gene gains (*i.e.*, the ‘Phytoplasma+’ set in Figure 2 and Table S2). Unfortunately, 46% of these genes are poorly characterized (COG categories R, S, and X) and it is difficult to infer the biological significance of these genes based on available annotation. Given the parasitic life cycle of these bacteria, it is possible that some of these poorly characterized genes may encode for proteins that phytoplasmas use to interact with their plant hosts or insect vectors [35], [36]. For example, several of the hypothetical proteins on this list (*e.g.*, YP_456212, YP_456572, YP_456673, etc.) were predicted to be secreted effectors or surface membrane proteins [37]. However, robust functional prediction based on sequence or conserved motif is difficult for these short and highly divergent hypothetical proteins. Nonetheless, by utilizing a phylogenetic framework to identify genes that are conserved among phytoplasmas but are absent in other related bacteria, our results have narrowed down the list of promising candidates for future empirical works to characterize their functions.

Other than the poorly characterized proteins, genes that are conserved among phytoplasmas but exhibit high levels of sequence divergence from other bacteria account for a substantial portion of the putative gene gains. These genes include several ribosomal proteins (COG category J, 19% of this gene set) and enzymes involved in the lipid biosynthesis (COG category I, 7% of this gene set). Although the presence of these genes cannot be considered as true gene gain, the driving forces behind this pattern of sequence divergence would be of interest for future molecular evolution studies.

Among the novel genes shared by all phytoplasma lineages and have good annotation, several appeared to have been introduced by potential mobile elements [8] or phages [38]. These genes often have multiple copies within each phytoplasma genome; examples include replicative DNA helicase (*dnaB*), DNA primase (*dnaG*), single-stranded DNA binding protein (*ssb*), ATP-dependent zine protease (*hflB*), and thymidylate kinase (*tmk*). Other notable examples include: (1) a P-type cation transport ATPase (*mgtA*), which may generate electrochemical gradient over the membrane and thus compliment the loss of F-type ATPases in phytoplasmas [8], (2) a Na+ driven multidrug efflux pump (*norM*), which may be involved in competition with other bacteria [39], and (3) a preprotein translocase subunit (*yidC*), which is involved in protein secretion [40] and likely to play a role in interaction with plant or insect hosts.

Compared to the hundreds of putative gene losses that were found in the common ancestor of Mollicutes, we identified only 27 putative gene losses in the common ancestor of phytoplasmas (*i.e.*, the ‘Phytoplasma−’ set in Figure 2 and Table S2). Two distinguished features include the losses of F_0_F_1_-type ATP synthase (*atpA*, *atpD*, and *atpG*) and pentose phosphate pathway (*pgcA*, *rpe*, *tkt*, *prs*, and *deoC*), which were reported in the initial genome analyses of phytoplasmas [8], [9]. In addition, several genes involved in purine salvage pathway (*apt* and *hprT*), pyrimidine metabolism (*trxB*), formylation of methionyl-tRNA (*fmt* and *folD*), protein degradation and modification (*clpC*, *lgt*, and *prkC*), biosynthesis of teichoic acid (*gtaB*), and potassium ion uptake (*ktrA* and *ktrB*) all appeared to have been lost early in the evolution of phytoplasmas. These results suggest the relaxation of selection for maintaining the related pathways in these obligate parasites and the process of genome degradation has continued after the evolutionary split between phytoplasmas and mycoplasmas. Interestingly, the phytoplasma-specific loss of an aspartyl/glutamyl-tRNA amidotransferase (containing two subunits: *gatA* and *gatB*) may have been complimented by the gain of a glutaminyl-tRNA synthetase (*glnS*) [41], [42].

### Mycoplasma-specific gene gains and losses

Our phylogenetic approach identified eight putative gene gains and eight putative gene losses in the common ancestor of the four mycoplasma lineages examined. Compared with phytoplasmas, the relatively low numbers of putative gene gains and losses may be explained by the high level of divergence among the mycoplasmas examined (see the branch lengths in Figure 1). Among these eight putative gene gains (*i.e.*, the ‘Mycoplasma+’ set in Figure 2 and Table S2), five are genes that show high levels of sequence conservation among mycoplasmas but are highly divergent from other bacteria (*atpB*, *ptsH*, *lip*, *yidC*, and *degV*). For example, another preprotein translocase (*yidC*) was identified as a putative gene gain in phytoplasmas and it bears no significant sequence similarity to the mycoplasma-specific *yidC*. The remaining three genes include a hexosephosphate transport protein (*uhpT*), a putative ATP-binding helicase protein, and a hypothetical protein.

Among the eight putative mycoplasma-specific gene losses we found (*i.e.*, the ‘Mycoplasma−’ set in Figure 2 and Table S2), three are considered to be artifacts due to high levels of sequence divergence among mycoplasma sequences. In other words, the corresponding genes from the eight non-mycoplasma genomes exhibit high levels of sequence conservation and are clustered in the same homologous gene cluster, whereas the mycoplasma genes are more divergent and thus scattered in several separate gene clusters. These genes include a cytosine deaminase (*codA*), a ribosomal protein (*rpsF*), and a translation factor (*sua5*). The remaining five true gene losses include the peptide chain release factor 2 (*prfB*, which corresponds to the modification of genetic code in *Mycoplasma*), a NAD-dependent malic enzyme (*sfcA*), two enzymes involved in tRNA modification (*cca* and *miaA*), and a primosome assembly protein (*priA*). Interestingly, the loss of this primosome assembly protein is observed in other sequenced mycoplasma genome [43] but this gene has been shown to be essential in *Bacillus subtillis* [44].

### Putative gene gains and losses in the outgroups

For the first outgroup (the class Clostridia, represented by *Clostridium kluyveri* and *Pelotomaculum thermopropionicum*), we identified 214 putative gene gains and three putative gene losses. However, assigning these events as putative gene losses and gains in the common ancestor of Mollicutes and Bacilli provides equally parsimonious explanations. Because we cannot be certain about the directionality of these changes and our main focus is on the gene content evolution in phytoplasmas and mycoplasmas, we choose not to over-interpret these two lists of genes.

For the common ancestor of the class Bacilli (represented by *Bacillus subtilis* and *Lactobacillus plantarum*), we identified 296 putative gene gains (*i.e.*, the ‘Bacilli+’ gene set in Figure 2 and Table S2) and no putative gene loss. However, because the taxon sampling in this study was designed to investigate the gene content evolution in phytoplasmas and mycoplasmas, this group of genomes is not ideal for characterizing the gene gains and losses in Bacilli. Thus, cautions should be taken in interpreting these results. Nonetheless, we found that genes involved in carbohydrate metabolism (COG category G), amino acid metabolism (COG category E), and transcription regulation (COG category K) are the three most abundant categories among the Bacillus-specific genes with specific functional annotation (accounts for 11%, 6%, and 6%, respectively; see Figure 2 and Table S2). This finding is consistent with the observation that Bacilli have versatile metabolisms that are under sophisticated regulations, which may have facilitated their expansion into diverse ecological niches.

## Discussion

By sampling an appropriate set of representative lineages and the utilization of a phylogenetic framework, our comparative analysis revealed intriguing patterns of gene gains and losses in two groups of important pathogenic bacteria. Our results suggest that the degradation of metabolic capacities in phytoplasmas and mycoplasmas has occurred predominately early in the evolution of Mollicutes, possibly associated with the transition to a host-dependent lifestyle. Furthermore, we identified a short list of genes that are conserved among sequenced phytoplasma genomes but are not present in other related bacteria. These genes may be good candidate for future experimental work to improve our understanding of how these parasites interact with their hosts. Importantly, the inference of a time interval for each putative gene gain or loss represents a major strength of our approach. Although the presence or absence of a particular gene or pathway may be apparent in the conventional pairwise comparisons between different genomes, establishing the timing and directionality of changes in gene content based on a phylogenetic framework is essential for understanding evolution.

The utility and reliability of our approach was demonstrated by the recovery of several key findings in previous studies, such as the loss of the F_0_F_1_-type ATP synthase and pentose phosphate pathway [9] and the gain of potential mobile elements [8] or phages [38] in phytoplasmas. However, despite the powerfulness of high-throughput large-scale comparative analyses, cautious examination of the results is indispensable. Because several factors can introduce complications in an analysis, naïve utilization of any bioinformatics pipeline can easily lead to erroneous conclusions. For example, specific patterns of sequence divergence can generate artifacts of gene gains or losses, such as the cases of putative gains of novel ribosomal proteins in phytoplasmas or the putative losses of other genes in mycoplasmas (see Results). In addition, the exact outcome of homologous gene clustering can be affected by the selection of genome sequences and the quality of annotation. For these reasons, careful manual curation is essential for extracting useful biological knowledge from a large-scale analysis like this.

Based on our current understanding of Mollicutes evolution, the group has evolved from a free-living ancestor approximately 590–600 million years ago [45]. Two major branches within this group, the AAA (*Asteroleplasma*, *Anaeroplasma*, and *Acholeplasma*; including phytoplasmas) and the SEM (*Spiroplasma*, *Entomoplasma*, and *Mycoplasma*), are thought to have diverged about 450 million years ago [45]. Although the reduction in genome size was hypothesized to have occurred independently in these two branches [45], our results suggest that the loss of metabolic capacities, particularly the biosynthesis of amino acids, nucleotides, and other metabolites, have occurred predominantly prior to the divergence between phytoplasmas and mycoplasmas. These changes are consistent with the expectation for the transition from a free-living to a host-associated lifestyle, as a large number of biosynthetic pathways became non-essential because many nutrients can be obtained from the host. In addition to the relaxation on selection to preserve genes involved in biosynthetic pathways, the reliance on hosts would also reduce the effective population size and increase the level of genetic drift for pathogenic bacteria [46], [47]. This increase in genetic drift, coupled with the strong mutational bias towards deletions observed in most bacterial genomes [48]–[51], appears to be the major driving force for genome reduction in the early evolution of Mollicutes. After the evolutionary split between phytoplasmas and mycoplasmas, the rate of genome reduction may have slowed down because the proportion of essential genes is relatively high in these already highly reduced genomes. This hypothesis is supported by the relatively few genus-specific gene losses observed in our results.

Although genome reduction has been a recurrent theme in pathogen evolution, acquisition of novel genes that the pathogens used to interact with their hosts is another important aspect. We identified a small list of hypothetical proteins that are putatively acquired by the common ancestor of phytoplasmas. Though the functions of these genes are currently unknown, the conservation of these sequences among genomes with a high propensity for gene losses is curious and may imply functional significance. Given the parasitic lifestyle of phytoplasmas, it is possible that at least some of these genes may be used for the interactions with their hosts [36], [52], [53]. For example, previous empirical studies have confirmed the role of several effectors encoded in the AYWB phytoplasma genome [37], [54]. Considering the laborious nature of experimental work on these important plant pathogens, our comparative approach is useful for the identification of promising candidate genes for future studies.

## Materials and Methods

### Data source and taxon sampling

To infer the gene content evolution in phytoplasmas and mycoplasmas, we obtained 12 complete genome sequences from NCBI GenBank [55] for comparative analysis. Detailed information about these 12 genomes, including the accession numbers, genome size, and other information, are provided in Table 1. This data set include all four available phytoplasma genomes, four representative *Mycoplasma spp.*, and two representative lineages each from Bacilli and Clostridia. Two major considerations in our taxon sampling include the phylogenetic distances among these lineages and the high quality of annotation available for each of these genomes. Although a large number of complete genome sequences are available from other *Mycoplasma spp.* and the two outgroups, the inconsistency in gene annotation across different genome sequencing efforts is likely to generate more false positive and false negative results in our definition of lineage-specific genes. For this reason, we employed this “representative lineage” approach instead of including all available genome sequences in this clade to achieve a balance between sensitivity and specificity.

### Homologous gene identification

To identify homologous genes among the selected genomes, we performed all-against-all BLASTP [56], [57] searches with an e-value cutoff of 1×10^−15^ for all annotated protein-coding genes. This choice of a stringent e-value cutoff prevents spurious hits between non-homologous genes that share some conserved domains and facilitates the identification of true homologous genes. The similarity results were supplied as the input for OrthoMCL [58] to perform homologous gene clustering. The algorithm is largely based on the popular criterion of reciprocal best hits between genomes for the identification of orthologous genes but includes additional normalization steps for between- and within-genome comparisons; an independent benchmarking study [59] has confirmed the reliability of this algorithm. All data parsing and processing steps were handled by a set of custom Perl scripts written with Bioperl modules [60].

### Inference of the organismal phylogeny

Based on the homologous gene identification result, we selected a set of single-copy genes shared by all genomes to infer the organismal phylogeny. Homologous gene clusters that contain more than one gene from any genome were not considered to avoid the complications introduced by paralogous genes in phylogenetic inference. For each homologous gene cluster, the protein sequences were aligned using MUSCLE [61] with the default settings. The resulting alignments of individual genes were concatenated to infer the organismal phylogeny using maximum likelihood, parsimony, and Bayesian methods.

For the maximum likelihood method, we used the program PhyML [62]. The amino acid frequencies, proportion of invariable sites, and gamma distribution parameter (with four categories of substitution rates) were estimated from the alignment in the maximum likelihood framework. To estimate the level of support for each internal branch, we generated 1,000 non-parametric bootstrap samples of the concatenated alignment by using the SEQBOOT program in the PHYLIP package [63] and repeated the phylogenetic inference as described above. For the parsimony approach, we used the program PROTPARS in the PHYLIP package [63]. To avoid the biases introduced by the input order of sequences, we enabled the jumble option to perform 1,000 randomization tests.

For the Bayesian approach, we used the program MrBayes [64], [65] to infer the posterior probability distributions of tree topologies and branch lengths with two independent runs. We enabled the mixed model option to sample all available amino acid substitution models and used four categories of substitution rates with a proportion of invariable sites for the gamma distribution. The Metropolis-coupled Markov chain Monte Carlo analysis was sampled every 500 generations for 1,000,000 generations with four chains in each independent run. The first 25% of the samples were discarded as the burnin process.

### Characterization of lineage-specific genes

Using the organismal phylogeny as the foundation, we categorized the homologous gene clusters according to the pattern of presence and absence in each of the selected genomes. Homologous gene clusters that can be explained by a single gene gain or loss events were counted and mapped on the phylogeny (see Figure 1). To check if the inferred gene losses were artifacts introduced by mis-annotation, we used all protein sequences in each homologous gene clusters as queries to perform TBLASTN [56], [57] searches against the complete genome sequences using a less stringent e-value cutoff of 1×10^−5^.

For functional categorization, all protein sequences were used as the query for a first-pass automatic annotation by utilizing the KAAS tool [66] provided by the KEGG database [67], [68]. The KEGG Orthology assignments were further mapped to the COG functional category assignment [69], [70] to generate summary statistics (see Figure 2). Genes that do have any COG functional category assignment were assigned to a custom category (category X: no COG assignment).

Finally, all results were manually inspected to examine the sequence similarity information (including the BLASTP and TBLASTN results), the original annotation provided in the GenBank records, the metabolic pathways involved, and additional information available from other databases [29], [30], [71], [72] and literature search.

## Supporting Information

Table S1 **Complete list of the 10,508 homologous gene clusters.**
(XLS)

Table S2 **Curated lists of putative gene gains and losses in the focal taxonomic groups.**
(XLS)
